# “Double awareness”—adolescents and young adults coping with an uncertain or poor cancer prognosis: A qualitative study

**DOI:** 10.3389/fpsyg.2022.1026090

**Published:** 2022-12-14

**Authors:** Vivian W. G. Burgers, Martin J. van den Bent, Judith A. C. Rietjens, Daniëlle C. Roos, Annemiek Dickhout, Suzanne A. Franssen, Marloes J. Noordoek, Winette T. A. van der Graaf, Olga Husson

**Affiliations:** ^1^Psychosocial Research and Epidemiology Department, Netherlands Cancer Institute, Amsterdam, Netherlands; ^2^Department of Medical Oncology, Netherlands Cancer Institute, Amsterdam, Netherlands; ^3^Department of Neurology, Erasmus University Medical Center, Rotterdam, Netherlands; ^4^Department of Public Health, Erasmus University Medical Center, Rotterdam, Netherlands; ^5^Department of Medical Oncology, Erasmus University Medical Center, Erasmus MC Cancer Institute, Rotterdam, Netherlands; ^6^Internal Medicine, Division Medical Oncology, Maastricht University Medical Center, Maastricht, Netherlands; ^7^GROW-School of Oncology and Reproduction, Maastricht University Medical Center, Maastricht, Netherlands; ^8^AYA Research Partner, Amsterdam, Netherlands; ^9^Department of Surgical Oncology, Erasmus University Medical Center, Erasmus MC Cancer Institute, Rotterdam, Netherlands; ^10^Division of Clinical Studies, Institute of Cancer Research, London, United Kingdom

**Keywords:** adolescents and young adults, uncertain or poor cancer prognosis, coping, qualitative research, CALM therapy

## Abstract

**Introduction:**

Adolescents and young adults with an uncertain or poor cancer prognosis (UPCP) are confronted with ongoing and unique age-specific challenges, which forms an enormous burden. To date, little is known about the way AYAs living with a UPCP cope with their situation. Therefore, this study explores how AYAs with a UPCP cope with the daily challenges of their disease.

**Method:**

We conducted semi-structured in-depth interviews among AYAs with a UPCP. Patients of the three AYA subgroups were interviewed (traditional survivors, new survivors, low-grade glioma survivors), since we expected different coping strategies among these subgroups. Interviews were analyzed using elements of the Grounded Theory by Corbin and Strauss. AYA patients were actively involved as research partners.

**Results:**

In total 46 AYAs with UPCP participated, they were on average 33.4 years old (age range 23–44) and most of them were woman (63%). Most common tumor types were low-grade gliomas (16), sarcomas (7), breast cancers (6) and lung cancers (6). We identified seven coping strategies in order to reduce the suffering from the experienced challenges: (1) minimizing impact of cancer, (2) taking and seeking control, (3) coming to terms, (4) being positive, (5) seeking and receiving support, (6) carpe diem and (7) being consciously alive.

**Conclusion:**

This study found seven coping strategies around the concept of ‘double awareness’ and showcases that AYAs are able to actively cope with their disease but prefer to actively choose life over illness. The findings call for CALM therapy and informal AYA support meetings to support this group to cope well with their disease.

## Background

Patients diagnosed with advanced cancer have to cope with a complex array of factors, including prolonged treatments, life-long side effects and a poor life perspective. These factors might be even more complicated in adolescents and young adults (AYAs; aged 18–39 years at primary cancer diagnosis) with an uncertain or poor cancer prognosis (UPCP). Most AYAs have not yet reached all milestones of an adult life and have less life experience to deal with their uncertain cancer diagnosis (García-Rueda et al., 2016; Bradford et al., 2022). A recent study shows that AYAs with a UPCP are confronted with ongoing and unique age-specific challenges, such as feeling inferior to previous self and others, sense of grief about life and not being able to make life plans (Burgers et al., 2022). This forms a tremendous burden and major life disruption for this young vulnerable patient group (Burgers et al., 2021, 2022). Given the “life-threatening” situation, that will likely not change during the rest of their lives, this group of AYAs must find a way to deal with cancer and its consequences for often a relatively long period.

While there is a growing body of research focusing on AYAs with advanced cancer, the current literature on how AYAs cope with cancer is mainly about older adult patients, patients with a relatively good prognosis or patients coping with nearing death. A recent study of Bradford et al. (2022), regarding AYAs with advanced cancer suggests that coping is a dynamic process where different strategies are used depending on the stressor, available resources and previous experiences. Earlier research on AYAs or adult patients with advanced cancer found acceptance, positive attitude by making comparisons with people in worse situations and setting realistic goals, keeping hope and support seeking as the most commonly used coping strategies (Trevino et al., 2012; Lobb et al., 2015; García-Rueda et al., 2016; Walshe et al., 2017; Lie et al., 2018; Bradford et al., 2022). Arantzamendi et al. (2020) redefined the theory of “living well with chronic illness” to explain the experience of living with advanced cancer. This new model involves an iterative process of struggling, accepting, living with advanced cancer, sharing the illness experience and reconstructing life, that occurs as the disease condition or the response to it change and people have to develop new ways to live well.

This redefined theory suggests that it might be possible to live well with cancer, but it requires deep engagement, time and effort (Arantzamendi et al., 2020). Up to now, little is known about the way AYAs living with a UCPC cope with their situation. Awareness of how these young people cope with daily challenges related to their UPCP is essential to understand how to improve the current care to empower young persons by strengthening individual coping styles and enhancing resources, with the ultimate goal to improve their quality of life. Therefore, this study explores how AYAs with a UPCP cope with the daily challenges of their disease.

## Materials and methods

This study presents a secondary analysis, a more detailed description of the methods can be found in Burgers et al. (2022). The Consolidation Criteria for Reporting Qualitative Studies guideline was followed to guarantee quality and transparency of reporting (Tong et al., 2007; Supplementary material 1).

### Participants

Patients were recruited from eight University Medical Centers in Netherlands, the Netherlands Cancer Institute and Haaglanden Medical Center. Eligible patients included those (1) diagnosed with any type of advanced cancer for the first time between 18 and 39 years of age, for which there is no reasonable hope of cure, indicating that the patient will die prematurely due to cancer, and (2) able to speak and understand Dutch. Patients with terminal illness with a life expectancy of less than three to 6 months and a poor performance status were excluded. Participating AYAs were classified into three groups based on received treatment and prognosis (Burgers et al., 2021). The three subgroups are: (1) AYAs treated with standard treatment(s) (mainly chemotherapy; traditional survivors); (2) AYAs undergoing novel treatment(s) (targeted therapy and immunotherapy; new survivors with uncertain prognosis); or (3) AYAs with a low-grade glioma (LGG) who are living with the knowledge that tumor progression inevitably occurs and likely will be lethal (Burgers et al., 2021). Additionally, we included five eligible AYA patients to participate in focus groups. The study obtained ethical approval by the Institutional Review Board of Netherlands Cancer Institute (IRBd20-205).

### Procedure

A trained female psychologist and qualitative researcher (VB) conducted all semi-structured interviews after informed consent. The interview guide was based on current literature and drafted in collaboration with experienced researchers (WG, OH) and AYA patients who were actively involved as research partners in this study (AD, NH, MH, SF, SF; Table 1). For more details on the active involvement of AYA research partners in this study, see the GRIPP2-SF checklist (Staniszewska et al., 2017) (Supplementary material 2). VB also led two online focus groups regarding the topic of coping in which preliminary results were discussed with AYAs to check for interpretation and gain additional insights. Interviews and the focus groups were audio-recorded and lasted on average 84 min (range: 49–122) and 55 min (range: 40–70) respectively. Besides the interview and focus group, patients completed a short case record form (CRF) on their sociodemographic and medical status. Additionally, clinical data was obtained from their treating clinician after patients gave explicit permission.

**Table 1:** Final version interview guide adolescents and young adult (AYA) interviews.

Questions	Probes
1. You have talked about how your disease has influenced your life. How do you currently cope with your disease and all associated challenges?	Could you please elaborate?
Could you please take me through a specific case which describes your experience in the best way? Could you please give me an example that showcases this well?	
2. What would be helpful for you to cope better with the situation?	Could you please elaborate? (what, when, how, with who)
3. Could you please provide me some words to describe the actual status of your disease right now?	Could you please explain to me wat this means?

### Data analysis

Interviews and focus groups were transcribed verbatim, and transcripts were analyzed by VB with the help of NVivo (QSR International Pty Ltd, 2022). Analysis was performed using elements of the grounded theory by Corbin and Strauss, such as open, axial and selective coding in combination with a constructivist philosophical perspective (Corbin and Strauss, 2014; Sebastian, 2019). A cyclic process was applied of interviewing, visualizing, analyzing and reflection by memo writing, leading to more specific questions. For the first 15 interviews (five each subgroup) were also analyzed by another independent researcher (MR) to identify and discuss different perspectives on the same data. In case of disagreement, the codes were discussed with the AYA research partners and the research team. Axial coding was done with the help of “the paradigm’ of Corbin and Strauss (2014). With selective coding all the data came together to construct an explanatory framework about coping with a UPCP at AYA age, which was checked by the research team and AYA research partners. Data collection stopped when conceptual saturation was reached (Corbin and Strauss, 2014).

## Results

### Demographics

In total, 64 patients were approached and 46 (72%) were actually interviewed. Eleven patients declined due to illness, four patients reported they were too busy or did not want to talk about their disease and three patients did not respond. The median age of the AYAs at time of the interview was 33.5 years and the majority of the participants were woman, had a partner and did not have children. Patients most frequently had (low-grade) gliomas, followed by sarcomas, breast cancer and lung cancer (Table 2).

**Table 2:** Demographics of AYA respondents and focus group participants.

Characteristics	Total AYAs^a^ *N* (%)	Traditional survivors *N* (%)	New survivors *N* (%)	LGG survivors *N* (%)	Focus group *N* (%)
Age at initial diagnosis, years					
Range	23–39	23–37	22–38	22–39	20–38
Mean (SD)	29.6 (4.8)	28.93 (3.9)	30.7 (4.9)	29.8 (5.7)	27.6 (6.6)
Current age, years					
Range	23–44	24–44	23–41	23–43	23–38
Mean (SD)	33.4 (6.3)	33.0 (5.6)	34.2 (4.8)	33.2 (5.6)	31.8 (5.5)
Gender					
Woman	29 (63.0)	12 (80.0)	10 (66.7)	7 (43.8)	4 (80.0)
Men	17 (37.0)	3 (20.0)	5 (33.3)	9 (56.3)	1 (20.0)
Other	0 (0.0)	0 (0.0)	0 (0.0)	0 (0.0)	0 (0.0)
Ethnicity					
Caucasian	46 (100)	15 (100)	15 (100)	16 (100)	5 (100)
Religion					
No	38 (82.6)	13 (86.7)	13 (86.7)	12 (75.0)	5 (100)
Yes^b^	8 (17.4)	2 (13.3)	2 (13.3)	4 (25.0)	0 (0.0)
Marital status					
Married or partnered	38 (82.6)	11 (73.3)	13 (86.7)	14 (87.5)	3 (60.0)
Single	8 (17.4)	4 (26.7)	2 (13.3)	2 (12.5)	2 (40.0)
Living situation					
Living alone	7 (15.2)	3 (20.0)	3 (20.0)	1 (6.3)	1 (20.0)
Living with partner	16 (34.8)	6 (40.0)	4 (26.7)	6 (37.5)	1 (20.0)
Living with partner and children	16 (34.8)	3 (20.0)	5 (33.3)	8 (50.0)	2 (40.0)
Living with children	3 (6.5)	2 (13.3)	1 (6.7)	0 (0.0)	0 (0.0)
Living with parents	2 (4.3)	1 (6.7)	0 (0.0)	1 (6.3)	0 (0.0)
Other^c^	2 (4.3)	0 (0.0)	2 (13.3)	0 (0.0)	1 (20.0)
Children					
Yes	19 (41.3)	5 (33.3)	6 (40.0)	8 (50.0)	2 (40.0)
No	27 (58.7)	10 (66.7)	8 (60.0)	8 (50.0)	3 (60.0)
Highest achieved level of education					
Secondary education or less	4 (8.7)	0 (0.0)	2 (13.3)	2 (12.6)	1 (20.0)
Secondary vocational education	16 (34.8)	5 (33.3)	4 (26.7)	7 (43.8)	1 (20.0)
Applied university	16 (34.8)	6 (40.0)	5 (33.3)	5 (31.3)	1 (20.0)
University	10 (21.7)	4 (26.7)	4 (26.7)	2 (12.5)	2 (40.0)
Employment status^d^					
Student	3 (6.5)	1 (6.7)	2 (13.3)	0 (0.0)	1 (20.0)
Full-time work	11 (23.9)	4 (26.7)	1 (6.7)	6 (37.5)	1 (20.0)
Part-time work	7 (15.2)	0 (0.0)	4 (26.7)	3 (18.8)	1 (20.0)
Self-employed	2 (4.3)	0 (0.0)	0 (0.0)	1 (6.3)	0 (0.0)
Unemployed	1 (2.2)	0 (0.0)	0 (0.0)	1 (6.3)	0 (0.0)
Homemaker	1 (2.2)	1 (6.7)	0 (0.0)	0 (0.0)	0 (0.0)
On sick-leave/ work disabled	23 (50.0)	9 (60.0)	7 (46.7)	6 (37.5)	2 (40.0)
Type of cancer					
(Low-grade) glioma	16 (34.7)	0 (0.0)	0 (0.0)	16 (100)	1 (20.0)
Sarcoma	7 (15.2)	6 (40.0)	1 (6.7)	0 (0.0)	1 (20.0)
Breast cancer	6 (13.0)	3 (20.0)	3 (20.0)	0 (0.0)	1 (20.0)
Lung cancer	6 (13.0)	0 (0.0)	6 (40.0)	0 (0.0)	1 (20.0)
Melanoma	3 (6.5)	0 (0.0)	3 (20.0)	0 (0.0)	0 (0.0)
Cervical cancer	2 (4.3)	2 (13.3)	0 (0.0)	0 (0.0)	0 (0.0)
Other^e^	6 (13.0)	4 (26.7)	2 (13.3)	0 (0.0)	1 (20.0)
Stage at diagnosis (interview)					
II	0 (30.4)	0 (0.0)	0 (0.0)	NA^f^	1 (20.0)
III	1 (3.3)	1 (6.7)	0 (0.0)	NA	0 (0.0)
IV	25 (83.3)	13 (86.7)	12 (80.0)	NA	2 (40.0)
N/A or unknown	4 (13.3)	1 (6.7)	3 (20.0)	NA	2 (40.0)
Current treatment^g^					
None/Active surveillance	16 (34.8)	4 (26.7)	1 (6.7)	11 (68.8)	2 (40.0)
Chemotherapy	14 (30.4)	9 (60.0)	2 (13.3)	4 (25.0)	1 (20.0)
Targeted therapy	10 (21.7)	0 (0.0)	10 (66.7)	0 (0.0)	1 (20.0)
Experimental therapy	3 (6.5)	0 (0.0)	2 (13.3)	1 (6.3)	0 (0.0)
Hormonal therapy	3 (6.5)	2 (1.3)	1 (6.7)	0 (0.0)	1 (20.0)
Immunotherapy	1 (2.2)	1 (6.7)	0 (0.0)	0 (0.0)	0 (0.0)
Radiotherapy	1 (2.2)	1 (6.7)	0 (0.0)	0 (0.0)	0 (0.0)
Years living with cancer					
Range	0–11	0–11	10–9	0–9	0–9
0–1	12 (26,1)	4 (26.7)	3 (20.0)	5 (31.3)	1 (20.0)
2–5	20 (63)	7 (46.6)	7 (46.7)	7 (43.7)	3 (60.0)
6–10	11 (24)	2 (13.3)	5 (33.3)	4 (25.0)	1 (20.0)
10+	2 (4.3)	2 (13.3)	0 (0.0)	0 (0.0)	0 (0.0)
Purpose of treatment according to AYA					
Prolong life	40 (89.1)	13 (86.7)	13 (86.7)	14 (87.5)	5 (100)
Cure	6 (10.9)	2 (13.3)	2 (13.3)	2 (12.5)	0 (0.0)
Comorbidity					
None	34 (74)	11 (73.3)	10 (66.7)	12 (75.0)	5 (100)
One	10 (21.7)	3 (20.0)	4 (26.7)	3 (18.8)	0 (0.0)
Two or more	2 (4.3)	1 (6.7)	0 (0.0)	1 (6.3)	0 (0.0)

^*^LGG = low-grade glioma. ^a^Total *N* = total interviewed AYAs, AYAs participated in focus groups are not included. ^b^Yes = Christian, Protestant, Islamic. ^c^Other = living with brothers, living with parents and son, living with housemates. ^d^Total exceed 100% since some participants were partly disabled and worked part-time for the other part. ^e^Other = (1) traditional survivors: colon cancer, epithelioid hemangioendothelioma, neuro-endocrine tumor, ovarian cancer. (2) new survivors: gastrointestinal stromal tumor. ^f^Low-grade gliomas are grade 2 tumors according to WHO classification of Tumors of the Central Nervous System. Two patients were initially diagnosed with a low-grade glioma, but at the time of the interview disease had progressed to a high-grade glioma grade III/IV. ^g^Not equal to 100% due to the combination of treatments.

### Findings

We identified seven main themes regarding coping with the daily challenges of AYAs living with a UPCP. The themes, sub-themes and quotes (including phase of AYA) are presented in Table 3. The numbers in the text refer to matching quotes of the AYAs to give a vivid illustration. Remarkable differences between AYA subgroups are presented in Table A1 in the Appendix.

**Table 3:** Overview of themes, sub-themes and quotes of AYA interviews.

Theme	Sub-theme	In-text-reference	Quotes
**1. COPING**			
Minimizing impact of cancer diagnosis	Focus on normal life	1.1	I actually attach a lot of value to the normal life, the daily routines of going to school and going to work. I really enjoy those things of a normal life. I also think that it shows that life goes on when you have cancer. (f, sarcoma, waiting phase)
	Life should not be defined by cancer	1.2	It is not the only thing that determines the choices I make, but of course it is a constant factor in every decision you have to make. But I try to think: “which decision would I have made if I did not have this tumor?” And then ask myself the question: “Now I do have a brain tumor, which choice do I make?” I try not to withhold myself from whatever decision I would have made when I was healthy. (m, low-grade glioma, phase unknown)
	Avoiding topic	1.3	Maybe it is a bit of superstition.. but I think if you are too busy with that [disease, consequences and death], it will come true. So I would rather not talk about death or anything. […] I am just very hopeful. (m, sarcoma, waiting phase)
Taking control and seeking certainty	Actively taking control on own health status	2.1	For me it is really about taking control, it gives me peace of mind that I know it [new treatment options] before my oncologist. I have a lot of confidence in my oncologist, but I do not believe that I am the first one that is on her mind when there is some new treatment on the market” (f, focus group)
	Arranging practical matters	2.2	My doctor said; “I do not think you should think about that yet [euthanasia].” But a week and a half later we were at the general practitioner to discuss euthanasia. I just want to be clear, it is about getting control I guess. For me it is like a plan that is on the bookshelf and I can pull it out when needed. It is just about being in control of my own life. (m, gastrointestinal stromal tumor, stable phase)
Coming to terms	No other choice than living with the cancer	3.1	I have been able to give it a place in my head but I am not willing to accept it. Yes, I have accepted that the cancer is in my life … to the extent that I accept that I do not do some activities anymore. I do not want to look back on my life too much. (m, melanoma, stable phase)
	Acceptation because of religion	3.2	I firmly believe that in the moment that we are created our death date is already predetermined. Only god knows how you will die. Will it be due to illness, will it be an accident? So I am not worrying about it.. when my day comes, it comes. You cannot escape it. (f, low-grade glioma, stable phase)
Positivity	Positive attitude	4.1	Someone else could end up under a bus tomorrow you know? So in that respect, that is how I approach life, no one is sure if he or she will make it. It could also be that a planet will bump into earth tomorrow and we are all death. (f, low-grade glioma, phase unknown)
	Keeping hope	4.2	I have heard about people who were going to die and are still alive ten years later. Why would it not be me? Why could I not be that exception? Maybe I keep too much hope for such a complicated disease. Once you stop hoping and stop working on the future, what are you doing it for? I am not going to throw in the towel. (m, sarcoma, waiting phase)
Seeking and receiving support	Emotional support	5.1	It is very nice to talk about this [cancer] with an external person. You can discuss it every day with your wife but you are kind of in the same place. It is very nice to have someone with a professional background who is trained for this and who can guide you in how to deal with emotions and how to best describe situations. And she also said: “Do not look too far ahead, because if your look too far ahead you always look at a place where you are not yet, so you are never satisfied. Live in the now and occasionally look back to see where you were and where you are now. Often enough you see improvement and that has a positive effect on your life.” This tip has helped me a lot. (m, low-grade glioma, stable phase)
		5.2	If I do not know what to do, I just ask Allah. That always gives me a boost. Also just praying is something spiritual, like going back to yourself. I do not know how to describe the feeling, but when you put your trust in Allah, you feel a certain peace in you and then you do not worry so much. (f, breast cancer, phase unknown)
	Relaxation	5.3	Everyday I try to meditate. In doing so, I also literally pronounce “I believe that I am going to cure.” In the beginning, it was really crazy to say but I notice that the more often I did it, the more I started to believe in it. I also believe that it can strengthen you and help you. At least by living longer. Furthermore, I really want to be alive and I think that helps me too. (f, sarcoma, phase unknown)
*Carpe diem*	Live in the moment	6.1	I surrender to it, it is what it is. You can worry about the things that would not work or make a lot of plans that ultimately need to be cancelled. Therefore we better just do fun things now, so you know you can enjoy the present. Because the treatments simply creates so much uncertainty and ambiguity. (m, sarcoma, rollercoaster phase)
	Speed up the fun things	6.2	I think we would have get married if we were older. This was not the time to get married, because I am still a student. So I wanted to work first and then got married, but now it goes a little faster because you do not know how much time you have left. (f, long cancer, stable phase)
	Easier to make choices	6.3	I live according to the traditional saying of “*carpe diem*.” There are certain things that I do not think about for too long. I am pretty impulsive myself, but I have become even more easy going. If I like it and I want to do it, then we are going to do it. I do not have to think about decisions that much anymore (m, low-grade glioma-stable phase)
Being consciously alive	Appreciating life	7.1	When the sun shines I can think… well it is nice weather, I am going for a walk with the dog. I never even thought about that before. You appreciate things more, especially small things. Before my diagnosis, I was like yes we go on a trip and on vacation and then we go there and then we plan a cruise and I want this and I want that. You always want everything but now I also just enjoy being at home with my family or going to the park. (f, melanoma, stable phase)
	Focus on the self	7.2	I put myself first now. I have to be really honest that I am much happier now than I used to be [before diagnosis]. I used to live in such a train that everything went on and on. It just feels nice this way, because I just choose more of what I find important. (f, cervical cancer, phase unknown)
	Changing priorities	7.3	Work has always come at the expense of my private life since I had no time and energy to see my friends or doing other things. So now I am not working anymore, it is very liberating. I now have much more peace than I ever had. I do not know if I would have allowed myself to quit that job without my illness. I think my life is fuller now than it was when I was not sick. If I continue my life without cancer I would have screwed up a very big part of my life. (f, breast cancer, stable phase)

#### Theme 1: Minimizing the impact of the cancer

Half of the AYAs with a UPCP want to minimize the impact of cancer by *focusing on living a normal life* in which they perform their daily routines like going to work and bringing their children to school. Living as normal as possible also includes not being identified as cancer patient, whereby AYAs try to keep up with their peers by ignoring physical complains and make sure that cancer is not always the center of attention. Many AYAs experience daily routines, fun activities and voluntary jobs as a good distraction to push the cancer to the background and live a “normal life” (1.1). Seeking distraction was challenging during the COVID-19 lockdown period. AYAs are confronted with many choices and decisions regarding their life and future plans without any future certainty. However, almost half of the AYAs reported their *life should not be defined by cancer* with the result that they make choices as if they are not ill, so they will not regret their decisions if they live longer (1.2). To avoid that life will be defined by the cancer, some AYAs did not want to know their prognosis at all. They are afraid that knowing their life expectancy will make them organizing their lives around a date of death. AYA men more often report this coping strategy. Another way AYAs try to minimize the impact of cancer is to *avoid cancer as topic*. Half of the AYAs reported that they are suppressing their thoughts and emotions regarding cancer and end of life since this causes negative emotions. Some AYAs associated thinking and talking about the cancer and its consequences as being weak and made them conscious of their poor prognosis, while others were still in the fighting mode and not ready to talk about it. At last, some AYAs believe they bring death upon themselves when focusing attention on this topic by talking about it (1.3). In general, minimizing the impact of cancer is most reported by traditional AYA survivors and AYAs with a low-grade glioma. However, AYAs indicate that minimizing the impact of the cancer does not mean they are completely in denial of their diagnosis.

#### Theme 2: Taking control and seeking certainty

Since uncertainty is overwhelming in AYAs with a UPCP, many try to take back any control or get any certainty where possible. This helps them to live as normal as possible. For example, by *actively taking control on own health status*. This includes; wanting to know every medical detail about their tumor or treatment and having control over treatment options (2.1). For some, understanding what is going on in their body and which treatments are available worldwide is a way to process the situation and to gain some hope. Other AYAs are trying to be in control by focusing on a healthy lifestyle, like exercising and specific diets. When they conduct this behavior, they are able to focus on their normal life routines. Furthermore, many AYAs are in need to take control regarding their *death and inheritance*. Having organized and planned euthanasia, the will and their funeral results in some kind of peace, allowing the AYA to move these topics to the background again (2.2). For some AYAs it is also about ensuring a desired future for their family by creating a lasting impact on the lives of their loved ones and making things easier on their family by leaving their partner and kids financially well.

#### Theme 3: Coming to terms

At least half of the AYAs report that they have come to terms with cancer and its consequences. A lot of them experience that there is *no choice other than living with the cancer* since fighting against it does not help and may make it even harder to live with cancer. Cancer is part of their lives and they have to find their way to live with it over time, however, not everyone can or will accept their situation (3.1). Some AYAs claimed they have accepted the cancer since this is how they simply deal with the situation or because of the comforting statements from their religion. A few AYAs find peace in their religion, for example they feel like they regain control over their lives by relinquishing control to Allah in which they are accepting Allah’s’ fate, suggesting their death date is predetermined (3.2). Relying on their faith was also helpful to make sense of one’s situation. All AYAs reported that time was helpful to find their way on how to deal to with their disease.

#### Theme 4: Positivity

The majority of the AYAs with a UPCP believe that a *positive attitude* is their motivational factor and has a positive impact on their mental and physical situation. This coping strategy is seen especially in new survivors. Some AYAs suggest having a natural positive attitude and others have learned to focus on the positive aspects of life. Another manner to be positive is by reframing. Like for example, reframing that sickness due to treatment is something positive since it is working against the cancer. Others are putting things into perspective, especially regarding the uncertainty in which they usually make the comparison that nobody is certain about their future (4.1). Many AYAs are keeping hope for a prolonged life or even a cure due to trust in medical science, the feeling of being a medical exception and feeling physically well (4.2). Keeping hope is the only way to keep them going. Coping with positivity is least used by AYAs receiving traditional treatment.

#### Theme 5: Seeking and receiving support

Almost all AYAs are seeking or receiving emotional support by family, partners, friends or AYA peers. Venting or doing fun things as a distraction feels supportive for AYAs. Others are seeking professional support by means of a psychologist, social worker or AYA clinical nurse specialist (5.1). Some AYAs seek religious support like praying to Allah (5.2). Nevertheless, several AYAs notice they are mostly supporting themselves, since they did not ask or receive the right support from others or felt like they do not fit in support groups. Others explained that they want to solve their problems on their own and want to experience the cancer process all by themselves, mostly in order to not burden others. Furthermore, relaxation methods like mindfulness and writing to express their feelings are also providing benefits for some AYAs (5.3).

#### Theme 6: Carpe diem

During the focus group sessions AYAs expressed that life was exhausting for them since they were always searching for some kind of balance in all the chaos. One AYA has found this new balance while the others are searching for it, asking themselves if they will ever find it. Some are actively searching for it while others are not being aware of this ‘new state of life’. Although this new state was not reported in the interviews, AYAs reported concepts of how to live relatively good with a UPCP. For most AYAs the context of an uncertain and poor prognosis results in *living in the moment*. They prefer to focus on living day by day instead of thinking about the future (6.1). Most of them learned it the hard way since planning the future is difficult, disappointing or sometimes not doable because of treatment and prognosis uncertainty. They try to enjoy the present to the fullest and *speed up the fun things* they would like to do in life, such as travelling and getting married (6.2). For some AYAs it is much *easier now to make choices* regarding things on their wish lists (6.3).

#### Theme 7: Being consciously alive

Being alive with a UPCP results in AYAs *appreciating* (the little) things in life more compared to before the cancer diagnosis, like enjoying the sun and being thankful for everyone around them (7.1). Some AYAs believe they now *focus more on themselves* instead of others (e.g., prioritizing friendships that give them energy), their job (e.g., quitting their job) or future (7.2). This is also the result of *changing priorities* since being aware of the value of life ensures AYAs to focus on their health and family (7.3). Some AYAs feel even happier and believe that their quality of live is increased.

## Discussion

**Figure 1:**
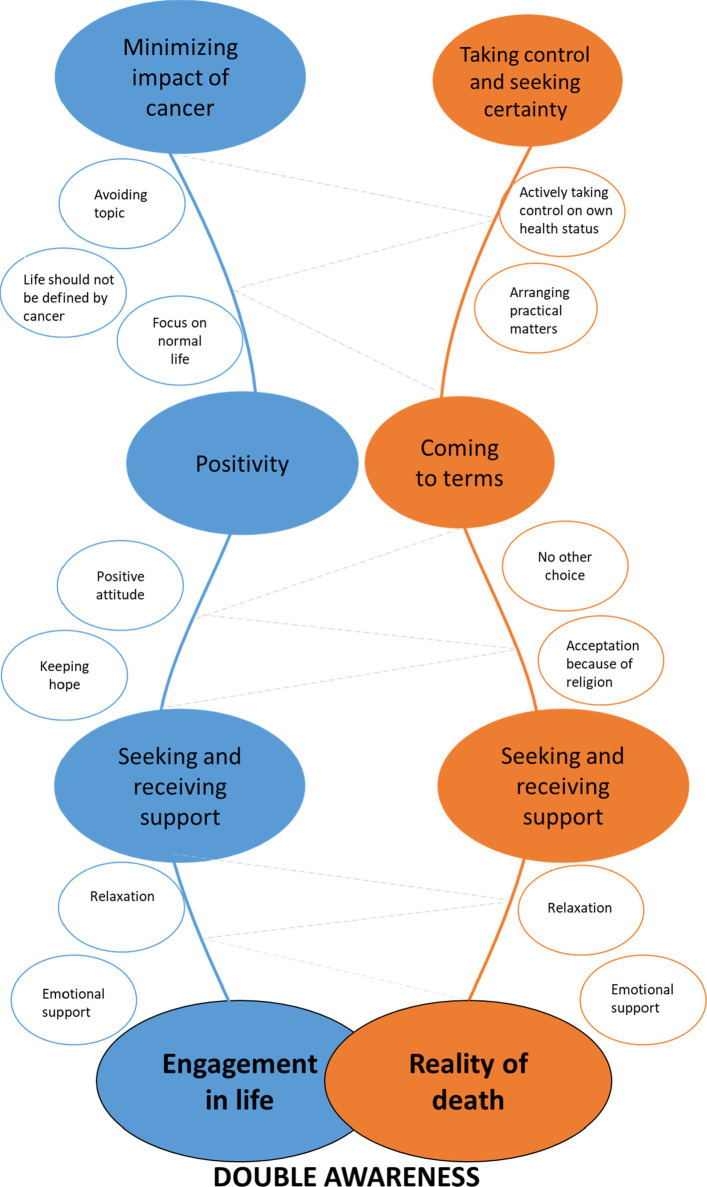
Double awareness in adolescents and young adults (AYAs) with a uncertain or poor cancer prognosis (UPCP). ^*^Size of the colored circles represents the frequency of the reported coping strategies. (e.g.) circles of ‘minimizing impact of cancer’ and ‘seeking and receiving support’ are largest, suggesting that these coping strategies were reported the most.

Findings from this qualitative interview study reveal that AYAs with a UPCP use multiple coping strategies like; minimizing impact of cancer, taking and seeking control, coming to terms, being positive, seeking and receiving support, *carpe diem* and being consciously alive in order to reduce the suffering from the experienced challenges. These results may indicate that this group is walking on two paths at the same time while coping with their UPCP; one path focusing on engagement in life and one path focusing on the reality of premature death. For example, some AYAs are hopeful for cure but at the same time accepting their poor prognosis. It seems that factors like; flow of life, hospital appointments or medical progression determine which path the AYA walks on. This capacity to cope in a context of a prolonged living-dying interval is earlier described by Rodin & Zimmerman as ‘double awareness’ (Rodin, 2013; Colosimo et al., 2018). See Figure 1 for the integration of double awareness in AYAs with a UPCP according to the results of this study.

With regard to the first path, the engagement in life, we found that AYAs are trying to *minimize the impact of cancer*. In line with previous research, AYAs want to stick to normalcy and seek distraction to avoid negative thoughts or situations that were anticipated to cause stress(Trevino et al., 2012; Walshe et al., 2017; Lie et al., 2018; Arantzamendi et al., 2020; Bradford et al., 2022). It is remarkable that coping by minimizing the impact of cancer was least reported by new survivors, possibly because these patients experience minimal impact of their treatment in daily life. It seems that especially men in this study do not want their life to be defined by cancer and implement strategies like not wanting to know their prognosis and not wanting to make other decisions than when they were healthy. Literature suggest that men wish to avoid engagement in issues of death and dying (Seifart et al., 2020). These observed gender differences might result from the product of a masculine gender-role in which men should be tough and emotionally inexpressive, which is easier when not being aware of the poor prognosis (Addis and Mahalik, 2003). Extra attention could be given in clinical practice to ensure that these AYA men also receive the right support. Another coping strategy we identified is *positivity*, which seems to be an essential manner of dealing with difficult emotions or situations and protecting mental health (Arantzamendi et al., 2020; Bradford et al., 2022). Previous research has found that positive framing was related to better quality of life and less depressive symptoms in incurable patients (Nipp et al., 2016). In line with these observations, hope seems to be a source of motivation for AYAs to face their present and future life (García-Rueda et al., 2016). The uncertainty in AYAs with a UPCP results in some extra options for hope, since there is no certainty of a poor end. It seems that AYAs on traditional treatments use positivity and hope less often, which could be explained by their poor health situation or by the fact that they did not have much hope that new treatments will arise.

Looking at the second path, reality of premature death, we found that AYAs want to have *control* over practical matters regarding death and inheritance, such as their funeral. To take back any control, AYAs want to actively take control of their own health status, which includes knowing all the details about their cancer diagnosis and focusing on new available treatment options. Half of the AYAs come to terms with their disease and get on with their lives. In line with previous research most participants described this as the only choice they had, because it was seen as essential to be able to move forward in life (Arantzamendi et al., 2020). Other research suggests that finding acceptance is correlated with less anxiety and depression and better quality of life (Nipp et al., 2016; Greer et al., 2020). At last, AYAs are *seeking and receiving emotional and professional support*. Earlier research suggested that social support is a key element in AYAs, resulting in higher psychosocial and existential quality of life and less grief (Trevino et al., 2012). These mental health benefits are also seen in correlation with religious coping, which helps AYAs to make sense of their situation in a way that supports acceptation (Alcorn et al., 2010; Barton et al., 2018; Arantzamendi et al., 2020). Young adults in the Dutch general population are described as less religious than older adults, however, religious young adults facing a disease view their religion as a guideline on how to cope with their disease and navigate their illness experience (King et al., 2021; CBS, 2022). AYAs also reported to seek social support, which is in contrast with our previous findings suggesting that AYAs feel alone since nobody truly understood their experiences and they tend to protect loved ones (Burgers et al., 2021). It is possible that having someone to count on does support the AYA, but is not the solution for the sense of loneliness.

Furthermore, theme six and seven of our result section suggest that AYAs with a UPCP strive to find a new balance in life (a new state) by living according to the metaphor *carpe diem* and being consciously alive. It could be that these patients have found double awareness. These results show some similarities with the study of Garcia et al. in which they identified that the majority of people with advanced cancer try to find (new) meaning in life to be able to enjoy life despite the disease (García-Rueda et al., 2016). In line with previous research it is about shifting the attention away from illness and the desire to live as fully as possible (Arantzamendi et al., 2020). Accepting the cancer and premature death facilitates this process. However, this process takes time and not everyone succeeds. Future research is needed to get a better understanding of this new state and examine predictive and risk factors.

### Strengths and limitations

This paper is the first to present how AYAs with a UPCP cope with their daily life challenges and offers insights in the combination of living well while also facing uncertainty around death. Additionally, this study is pioneering in involving patients as AYA research partners. In terms of qualitative research, the sample size is relatively large. This study also has some limitations. First, the representation of ethnic minority groups was limited. Since religion seems to play an important role in coping with a UPCP, future research should especially focus on the coping style of ethnic minority groups. In our sample we did not observe important differences in coping strategies between different ages. However, it should be mentioned that young AYA patients between the ages of 18 and 25 were slightly underrepresented. Lastly, according to the concept of double awareness, coping strategies may fluctuate over time. Since this study is cross-sectional, we were not able to access this process. A longitudinal study is recommended to examine the fluctuating process of coping over time.

### Clinical implications

Taking into account the positive correlation between coping strategies and quality of life, it seems indeed possible to live well with a UPCP. However, we have to keep in mind that some AYAs are still struggling to cope with the disease and not everyone reached double awareness. For example, AYAs can be in a problematic relation with the idea of death, such as avoiding making plans due to preoccupation with mortality (Burgers et al., 2021). When experiencing this tensions between life and death, Managing Cancer And Living Meaningfully (CALM) therapy, could be recommended. This brief, tailored psychotherapeutic intervention seems effective in relieving and preventing depressive symptoms and can help patients to address preparations for the end of life (Rodin et al., 2018). Colosimo and colleagues provide a framework for using CALM to cultivate double awareness (Colosimo et al., 2018). However, up to now, there is no literature focusing on CALM therapy in AYA patients and the question that arise is how double awareness works in this AYA group given their unique age, developmental phase of life and prognostic characteristics of their disease. Future research on the concept and determinants of double awareness in AYA patients with a UPCP, and the effectiveness of CALM therapy in this patient group is needed. Furthermore, the positive effects of social support, specifically AYA peer support, seems not always as effective as described in literature. In practice, some AYAs want to avoid negativity and confrontation and therefore do not want to join a peer support group (Bradford et al., 2022). Since they may still have the need for mutual interactions, AYA peer support should also offer the option of informal meetings. For example, the Dutch AYA ‘Young and Cancer’ Care Network has implemented AYA lounges in hospitals and the Dutch Youth and Cancer foundation is organizing meetings in bars. At last, a positive attitude or minimizing the impact of cancer by preferring not to know prognosis or medical information is not directly an indication of lack of awareness of dying. Instead, it could be a protective function as it allows hope (Hagerty et al., 2005). We encourage healthcare professionals to explore patients coping style and in specific prognostic information preferences and the underlying reasons in order to provide tailored communication (van der Velden et al., 2022).

### Conclusion

We identified seven different coping strategies around the concept of ‘double awareness’ and found that AYAs want to reach a new balance in life in which they manage their new state. AYAs are able to actively cope with their UPCP but actively choose life over illness and also need a break from cancer sometimes. However, this does not imply that they deny the cancer and its consequences. Recommendations included CALM-therapy and informal AYA support meetings targeted to UPCP to support AYAs to cope well with their UPCP.

## Data availability statement

The raw data supporting the conclusions of this article will be made available by the authors, without undue reservation.

## Ethics statement

The studies involving human participants were reviewed and approved by Institutional Review Board of Netherlands Cancer Institute (IRBd20-205). The patients/participants provided their written informed consent to participate in this study.

## Author contributions

OH and WG conceptualized the study and acquired funding. VB, AD, SF, MN, WG, and OH developed methodology. VB, MB, DR, and WG contributed to patient recruitment. VB performed interviews and analysis with help of AD, SF, MN, WG, and OH were also involved in analysis discussions. The original draft was prepared by VB, WG, and OH. All authors contributed to the article and approved the submitted version.

## Funding

OH and VB are supported by a grant from the Netherlands Organization for Scientific Research [grant number VIDI198.007].

## Conflict of interest

The authors declare that the research was conducted in the absence of any commercial or financial relationships that could be construed as a potential conflict of interest.

## Publisher’s note

All claims expressed in this article are solely those of the authors and do not necessarily represent those of their affiliated organizations, or those of the publisher, the editors and the reviewers. Any product that may be evaluated in this article, or claim that may be made by its manufacturer, is not guaranteed or endorsed by the publisher.

## Appendix 1

**Table A1:** Themes and sub-themes with remarkable differences between AYA subgroups.

Themes, sub-themes, codes	Less often reported by	%	More often reported by	%
1. Minimizing impact of the cancer diagnosis	new survivors	73	traditional survivors/low-grade glioma	100/94
1.1. Focus on normal life	new survivors/low-grade glioma	40/44	traditional survivors	67
1.2. Life should not be defined by cancer	woman	37	Men	53
1.3. Avoiding cancer as topic	new survivors/traditional survivors	47/53	low-grade glioma	73
				
2. Taking control and get certainty	low-grade glioma/traditional survivors	44/53	new survivors	80
2.1. Arranging practical matters	low-grade glioma/traditional survivors	27/27	new survivors	67
2.2. Actively taking control on own health status	Men low-grade glioma/traditional survivors	26 23/33	woman new survivors	45 67
				
4. Positivity	traditional survivors	53	low-grade glioma/new survivors	88/87
4.1. Keeping a positive attitude	traditional survivors/low-grade glioma	33/44	new survivors	73
				

*We cannot claim that differences reported in this table are satistical significant. Percentages are based on: n reported/total n of subgroup (e.g. men reported subtheme 1.2/total amount of men).
