# Supplementary material for: “Double awareness”—adolescents and young adults coping with an uncertain or poor cancer prognosis: A qualitative study
Source: Front Psychol. 2022 Dec 14;13:1026090. doi: 10.3389/fpsyg.2022.1026090 (PMC9795247; doi:10.3389/fpsyg.2022.1026090)
Supplement: Supplementary file 2 [file Data_Sheet_2.PDF]

Supplement 2. GRIPP-2 Checklist

Section and topic	*Item*	*page No*
1: Aim<br>Report the aim of the study	The aim of this study was to explore how AYAs with an uncertain or poor cancer prognosis (UPCP) cope with the daily challenges of their disease.	p. 2
2: Methods<br>Provide a clear description of the methods used for PPI in the study	Five AYAs with UPCP were actively involved as AYA research partners in this study. One AYA was already involved in the phase of grant application and recruited another AYA, two other AYAs were invited to contribute as research partner after the participated in the interview study and one AYA was recruited via the researcher. According to each preferences and availability, AYA research partners attending several online meetings to contribute to this study.	p. 3
3: Study Results<br>Outcomes—Report the results of PPI in the study, including both positive and negative outcomes	AYA research partners contributed to the study in several ways, including:<br>- Being a participant in the pilot testing phase<br>- Reviewing and adapting the patient information letter for relevance, word use and level of confrontation<br>- Reviewing and adapting the interview guide for relevance, comprehensiveness, word use and level of confrontation<br>- Discussing about how to provide the best aftercare for participants<br>- Contributing in consensus meetings about the results and checking for correct interpretation<br>- Checking the general framework for misinterpretation or gaps in logic<br>- Editing and being co-author of the paper	p. 3
4: Discussion and conclusions<br>Outcomes—Comment on the extent to which PPI influenced the study overall. Describe positive and negative effects	Collaboration with AYA research partners provides more appropriate and relevant research regarding AYAs with a UPCP and contributed to a better translation to suggestions for clinical practice. Adjustment and prioritisation of interview questions, information about appropriate questions and knowledge about the wish for after care, resulted in an improved methodology and more confidence in the researcher. During the discussion of the results, themes and sub-themes were renamed and the figure was adapted. The experience of the AYA research partners also provides more insight and context to interpret the results of the study. The AYA themselves were proud to contribute, appreciated the feeling of being seen of added value and received support from the contact moments with other AYA research partners.	N/A
5: Reflections/critical perspective<br>Comment critically on the study, reflecting on the things that went well and those that did not, so others can learn from this experience	Patient involvement is an investment of time and effort, since you have an extra group of people who have to make decisions about your study. These research partners are included because of their experiential knowledge and not because of their scientific skills, which can result in the need for extra coaching and information. This also requires some extra skills of the researcher. The main challenge but also the most important aspect is regularly evaluating and staying in contact with the AYA research partners. Even when they are not that involved at that moment (e.g. writing manuscript). As conclusion, seeing the impact of performing research with AYA instead of about AYA is worth the investment. Timely preparation and good communication can make it less time consuming.	-N/A
